# An Interesting Case of Macular Amyloidosis With No Significant Etiology

**DOI:** 10.7759/cureus.56248

**Published:** 2024-03-16

**Authors:** Avni Gakkhar, Ashok M Mehendale, Shivansh Mehendale

**Affiliations:** 1 Preventive Medicine, Jawaharlal Nehru Medical College, Datta Meghe Institute of Higher Education and Research, Wardha, IND

**Keywords:** itching, scrub, radiation, friction, skin, plca, ma

## Abstract

Macular amyloidosis is primary localized cutaneous amyloidosis (PLCA). It is described by the extracellular accumulation of heterogenic amyloid proteins in the skin that does not affect the systemic immune system, causing hyperpigmented patches. It is a prevalent skin disorder of young female adults, especially in India, since it affects the population with darker skin. History of frictional rub on the skin is typically present, such as using loofah or bathing scrubs or stones. The case presented below is of a 23-year-old female who presented with a hyperpigmented patch on the upper back of both sides and extensor surface of arms and did not have any history of usage of loofah on those areas, compelling us to research more on the other causative factors (genetic predisposition, infectious agents, and UV radiation are probable causative factors) for macular amyloidosis. This condition is not entirely cured; it is managed symptomatically only to improve cosmetic outcomes.

## Introduction

Macular amyloidosis (MA) is a disease that is less understood. Usually presents as grey-brown, itchy macules, MA eventually unites into ripple-patterned patches on the upper back, more commonly on the interscapular area and the extensor aspect of the arms, chest, and thighs [1]. It is more common in the Asian population with a predisposition to young adults with darker skin, and a preponderance to the female sex is present. Dryness of skin, temperature of living area, and use of irritants on the skin are also factors that play a role. MA is the most prevalent form of primary localized cutaneous amyloidosis (PLCA). Lichen, MA, and nodular amyloidosis are the three principal PLCA types [2]. PLCA is limited to the skin and less likely to produce systemic manifestations, but still, a few diseases are associated with it and listed later in the report. In MA, keratinocytes produce the amyloid deposits. Repeated trauma to the skin in the form of friction leads to keratinocyte damage that leads to the production of an eosinophilic, amorphous substance called amyloid. Although the precise pathophysiology causing keratinocytes to undergo apoptosis is unknown, friction (rubbing or scratching), genetic susceptibility, pathogens, and UV radiation are potential sources of trauma to the skin [1,2]. Diagnosis is mainly made clinically, but confirmative diagnosis is made by biopsy [3]. Amyloid can be seen as eosinophilic deposits, dermal melanophages accompany the extracellular material deposited in the papillary dermis seen on Hematoxylin and eosin-stained skin. There is apple-green birefringence on Congo red staining under polarized light [4]. It is necessary to confirm the presence of amyloid to distinguish other skin conditions from MA. Laboratory investigations are of no value in amyloidosis. No treatment therapy is standard since there is no cure for this condition, and the patient is treated symptomatically. Anything that causes frictional rub on the patient's skin should be prevented; if the patient is on any immunosuppressive drug, it should be stopped, and radiation exposure should be avoided in genetically predisposed patients. Medications are topical steroids, cyclosporine, and topical calcineurin inhibitors including tacrolimus [5]. Physical modalities like Ultraviolet (UV) B phototherapy (narrow band and broadband) and PUVA (psoralen with UVA) phototherapy are used when medications do not work. The surgical options include CO_2_ laser therapy, cryosurgery, and dermabrasion [3,4].

## Case presentation

**Figure 1:**
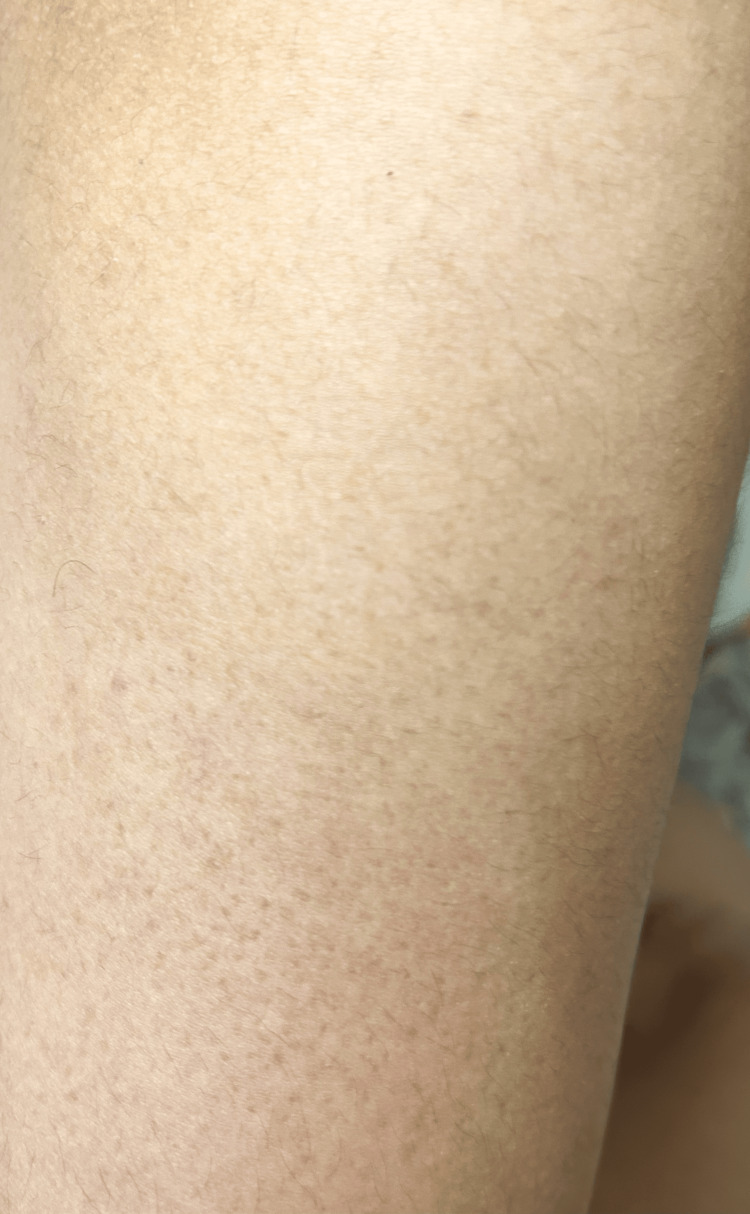
Macular amyloidosis on extensor surface of right arm

**Figure 2:**
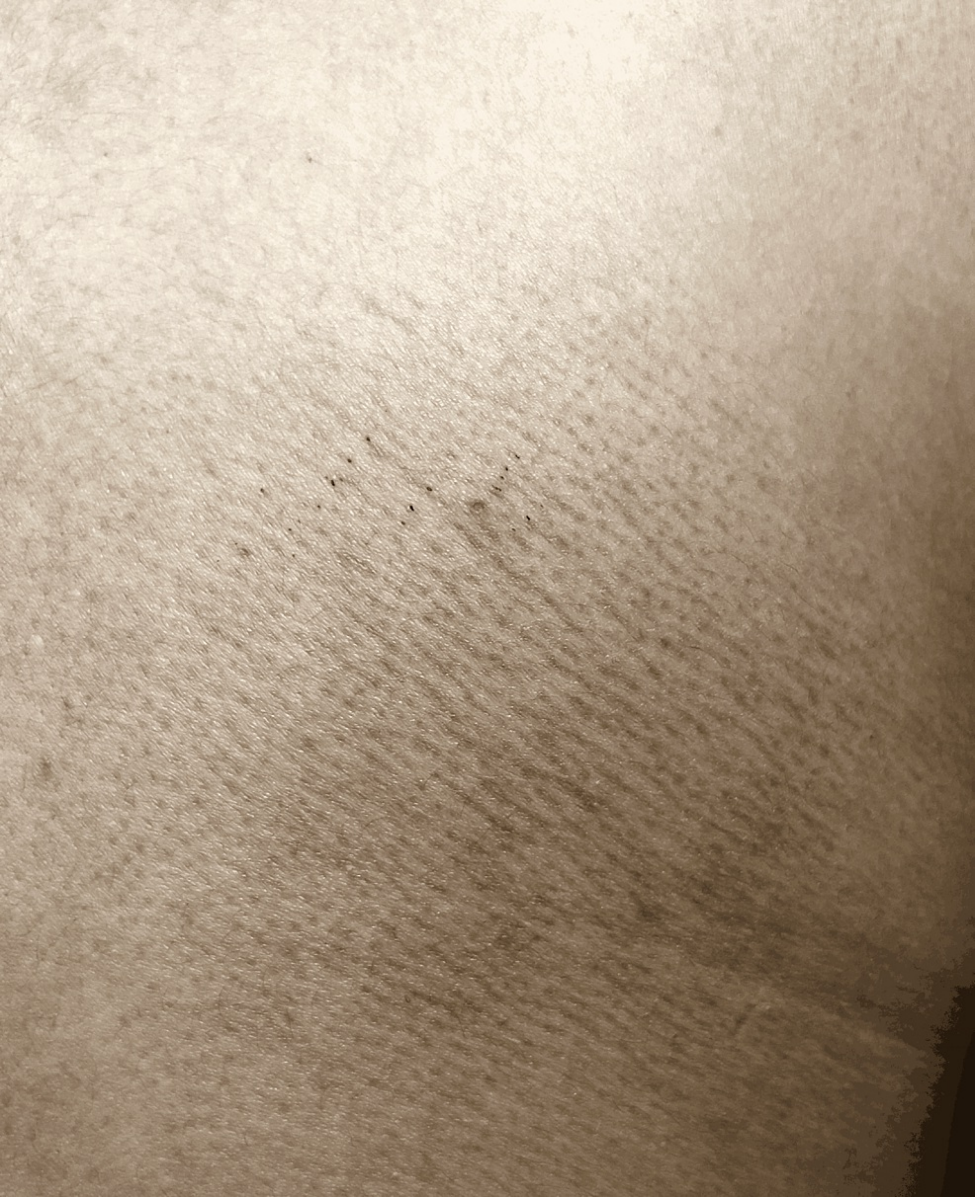
Macular amyloidosis on scapular region of back in right side

**Figure 3:**
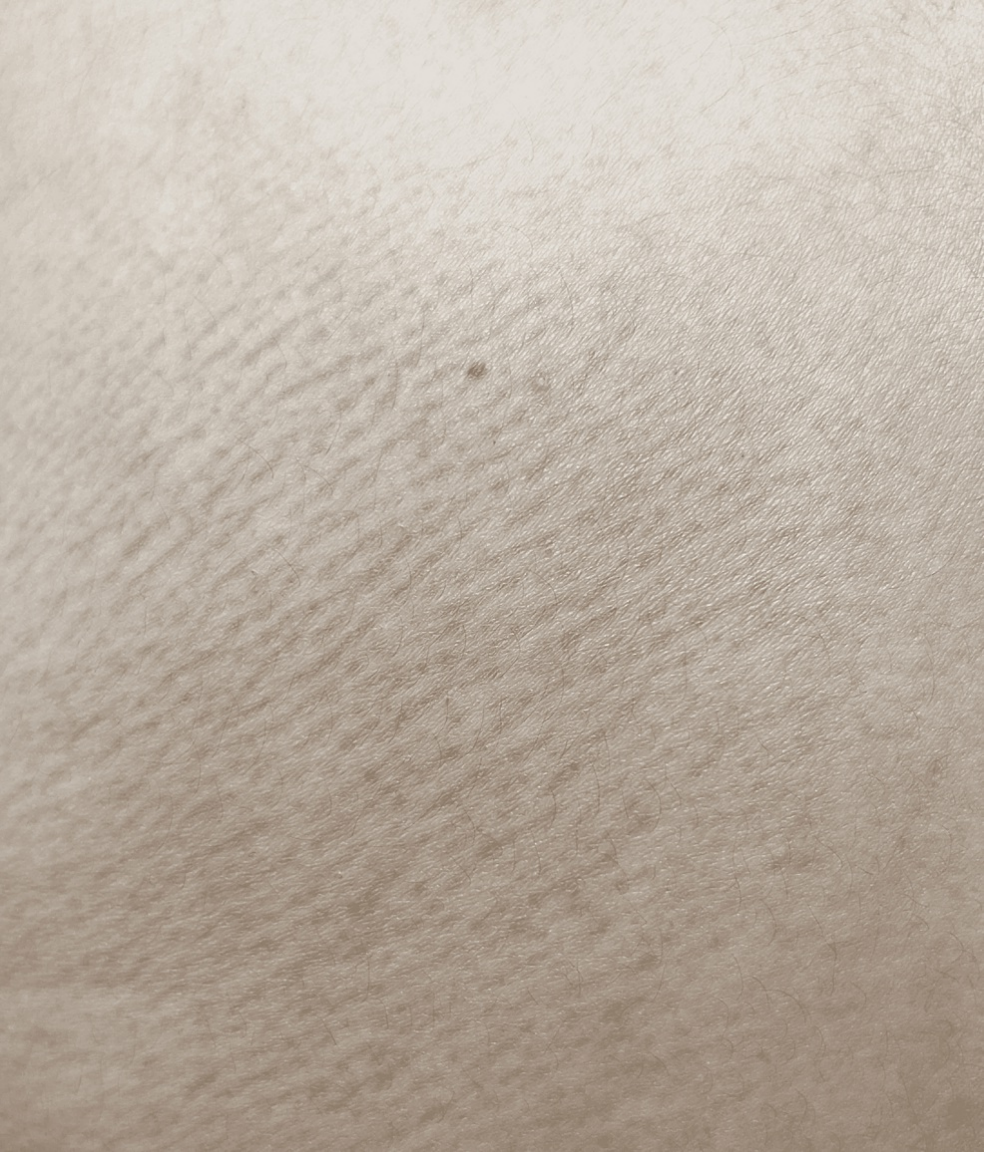
Macular amyloidosis on scapular region of back in left side

**Figure 4:**
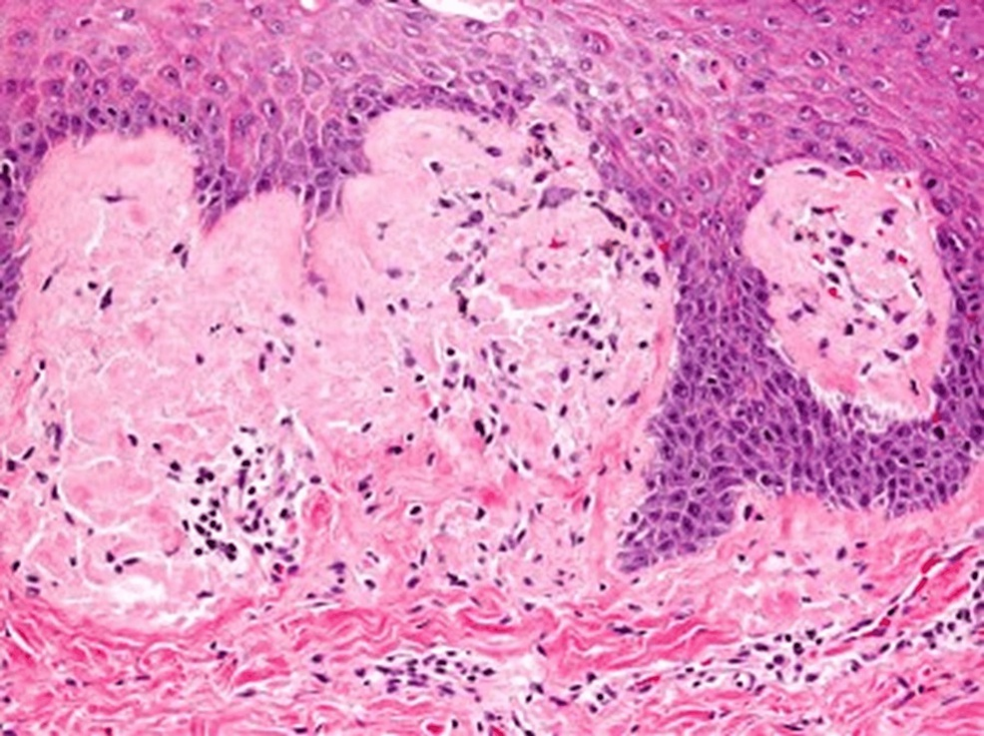
Biopsy of the lesion shows macular amyloidosis in the dermis.

A 23-year-old female presented with pruritic and hyperpigmented lesions on the upper back in scapular regions on both sides and the extensor surface of both arms. The patient has a history of having dry skin, lives in an area with a hot and humid climate, and uses non-medicated moisturizer on the skin. It was not associated with nodule formation, inflammation, pus, or fever. There was no history of loofah use, scrubs, other skin irritants, or any drug application. Lab investigations were done and were normal. Figures 1-3 show MA on the extensor surface of the right arm and the scapular region of the back on the right and left sides, respectively. A lesion biopsy was performed (Figure 4), which showed lymphocytic infiltration and amorphous deposits in the dermis; hence, the clinical diagnosis of MA was confirmed. Medications prescribed were 0.1% Topical Tacrolimus application for 30 mins, rinse with a warm water-soaked towel (avoiding rubbing), and emollient use afterward. Cream Demelan was also prescribed to be applied twice daily. The patient was relieved, and the lesions stopped progressing.

## Discussion

The causation cannot be restricted to friction on the skin; gender, race, genetic predisposition, UV radiation, allergy, and auto-immunity have all been linked to increased risk. It has been proposed that female predominance is related to women seeking medical assistance earlier for visually undesirable pigmentation [6]. Female sex hormones also play a role and are researched, but conclusive reports and research are still lacking [4]. Hyperpigmentation, which can occur in vast regions of the back, particularly the interscapular region, arms, and legs, is a significant cosmetic issue. Overall, the treatment of PLCA is poor. Mild cases can be treated with topically applied corticosteroids, either with or without occlusive dressings, and photoprotection [7]. A topical application of 10% dimethyl sulphoxide (DMSO) is recommended, although the outcomes have been mixed. UVB therapy has also been used successfully to treat macular and papular amyloidosis. Etretinate and acitretin therapy have been effective in some cases, although the illness appears to recur once treatment is discontinued. Cyclophosphamide, cyclosporine, and dermabrasion are treatment methods with minimal therapeutic effectiveness [5-8]. The Q-switched Nd-YAG laser (532 nm and 1064 nm) has demonstrated promising results in decreasing pigmentation in MA. A newly identified cytokine, interleukin (IL)-31, has been postulated to play a role in relieving the problem of itching/pruritis and has been linked to the pathophysiology of itchy dermatoses such as PLCA (familial and sporadic) [6]. This idea has prompted investigation into newer pharmaceutical therapeutics targeting the IL-31 receptor in treating PLCA and other itchy dermatosis [9]. MA is limited to only amyloid deposition in the skin and does not progress to systemic diseases. However, there is evidence that PLCA is associated with several immune disorders, including systemic sclerosis, CREST syndrome, rheumatoid arthritis, systemic lupus erythematosus, primary biliary cirrhosis, IgA nephropathy, and sarcoidosis [1]. The need for defined etiological factors makes it challenging to recommend a suitable therapy modality [4,10].

## Conclusions

MA is a common skin condition among Asians and in India, young age of onset, female preponderance, early onset in women, greater involvement of the upper back and extensors of the arms, and itching symptoms. Radiations due to exposure to the sun have a significant impact on illness localization. The remark on the role of friction on the skin could not be definitively supported or refuted. The need for a defined etiological factor makes recommending a suitable therapy modality difficult. Due to a lack of understanding of the disease's natural course and clear cause, MA remains a mystery and source of concern for the world. Since the etiology is not entirely understood, there is no standard treatment, but preventing UV exposure, allergens on the skin, and physical skin irritants are helpful.

## References

[1] (2024). Macular amyloidosis (friction Amhyloidosis) - dermatology advisor. https://www.dermatologyadvisor.com/home/decision-support-in-medicine/dermatology/macular-amyloidosis-friction-amhyloidosis/.

[2] Weidner T, Illing T, Elsner P (2017). Primary localized cutaneous amyloidosis: a systematic treatment review. Am J Clin Dermatol.

[3] Chia B, Tan A, Tey HL (2014). Primary localized cutaneous amyloidosis: association with atopic dermatitis. J Eur Acad Dermatol Venereol.

[4] Hudson LD (1986). Macular amyloidosis: treatment with ultraviolet B. Cutis.

[5] Bandhlish A, Aggarwal A, Koranne RV (2012). A clinico-epidemiological study of macular amyloidosis from north India. Indian J Dermatol.

[6] Ostovari N, Mohtasham N, Oadras MS, Malekzad F (2008). 532-nm and 1064-nm Q-switched Nd:YAG laser therapy for reduction of pigmentation in macular amyloidosis patches. J Eur Acad Dermatol Venereol.

[7] Zhang Q, Putheti P, Zhou Q, Liu Q, Gao W (2008). Structures and biological functions of IL-31 and IL-31 receptors. Cytokine Growth Factor Rev.

[8] Sonthalia S, Agrawal M, Sehgal VN (2021). Dermoscopy of macular amyloidosis. Indian Dermatol Online J.

[9] Barroso B, Torrico-Hatami S, Otal-Buesa M, Bernaola J, Haro R (2022). Macular amyloidosis in an atopic patient after aeroallergen immunotherapy. J Allergy Clin Immunol Pract.

[10] Chuang YY, Lee DD, Lin CS, Chang YJ, Tanaka M, Chang YT, Liu HN (2012). Characteristic dermoscopic features of primary cutaneous amyloidosis: a study of 35 cases. Br J Dermatol.

